# The Effectiveness of a Structured Educational Intervention on Knowledge, Beliefs, and Self‐Reported Practices of Pregnant Women Exposed to Thirdhand Smoke: A Randomized Controlled Trial

**DOI:** 10.1002/brb3.71330

**Published:** 2026-06-08

**Authors:** Nooshin Yoshany, Zahra Afkhami, Mahsa Khodayarian, Sara Jambarsang, Amir Ghaderi, Zohreh Karimiankakolaki

**Affiliations:** ^1^ Department of Health Education and Promotion, Social Determinants of Health Research Center, School of Public Health Shahid Sadoughi University of Medical Sciences Yazd Iran; ^2^ Department of Health Education and Promotion Social Determinants of Health Research Center, School of Public Health Shahid Sadoughi University of Medical Sciences Yazd Iran; ^3^ Social Determinants of Health Research Center, Department of Health Education & Health Promotion, School of Public Health Shahid Sadoughi University of Medical Sciences Yazd Iran; ^4^ Center For Healthcare Data Modeling, Department of Biostatistics and Epidemiology, School of Public Health Shahid Sadoughi University of Medical Sciences Yazd Iran; ^5^ Social Determinants of Health (SDH) Research Center Kashan University of Medical Sciences Kashan Iran; ^6^ Clinical Research Development Unit‐Matini/Kargarnejad Hospital Kashan University of Medical Sciences Kashan Iran; ^7^ Social Determinants of Health Research Center, Shk.C. Islamic Azad University Shahrekord Iran; ^8^ Department of Health, Shk.C. Islamic Azad University Shahrekord Iran

**Keywords:** beliefs, health education, knowledge, pregnant women, self‐practices, thirdhand smoke

## Abstract

**Background:**

Thirdhand smoke (THS) is a persistent environmental toxin that poses significant health risks, especially to vulnerable populations such as pregnant women and their fetuses. Effective interventions to enhance knowledge, modify beliefs, and improve protective practices are crucial. This study aimed to evaluate the impact of a structured, multi‐component educational intervention on knowledge, beliefs, and self‐reported practices related to THS among pregnant women.

**Methods:**

A randomized controlled trial was conducted with 100 pregnant women in their second or third trimester, recruited from comprehensive health centers in Isfahan, Iran. Participants were randomly assigned to an intervention group (n = 50) or a control group (n = 50). The intervention group received a multi‐faceted educational program consisting of four face‐to‐face group sessions, one spousal session, educational pamphlets, and reinforcement messages via a social media group. The control group received routine prenatal care. Data were collected using validated questionnaires assessing knowledge (11 items), beliefs (using the Beliefs About Thirdhand Smoke—BATHS scale, 9 items), and self‐reported practices (9 items) at baseline and two months post‐intervention. Data were analyzed using paired t‐test, Wilcoxon signed‐rank test, and Analysis of Covariance (ANCOVA) in SPSS v.23.

**Results:**

The intervention group demonstrated significant improvements in mean scores of knowledge (from 13.30 ± 2.68 to 21.52 ± 5.23, *p* < 0.001), beliefs (from 25.60 ± 8.12 to 38.46 ± 4.01, *p* < 0.001), and self‐reported practices (from 10.50 ± 1.50 to 13.52 ± 2.10, *p* < 0.001) after the intervention. No significant changes were observed in the control group. ANCOVA results, while controlling for baseline scores, confirmed a statistically significant effect of the intervention on post‐intervention scores for knowledge (F = 179.61, *p* < 0.001), beliefs (F = 77.52, *p* < 0.001), and practices (F = 54.14, *p* < 0.001).

**Conclusion:**

A structured, theory‐based educational intervention significantly improved knowledge, beliefs, and self‐reported protective practices concerning THS among pregnant women. Integrating such comprehensive programs into routine prenatal care is strongly recommended to mitigate THS exposure risks and promote maternal and fetal health.

**Trial Registration:**

This randomized controlled trial was prospectively registered in the Iranian Registry of Clinical Trials (IRCT) on 25/04/2025, prior to participant recruitment (Identifier: IRCT20250105064282N1).  https://irct.behdasht.gov.ir/trial/81695.

## Graphical abstract

Change in knowledge, beliefs and self‐reported practice of pregnant women exposed to third hand smoke

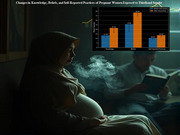

**Abbreviations**
ANCOVA: Analysis of Covariance
CVI: Content ValidityIndex
CVR: Content Validity Ratio
RCT: Randomized Controlled Trial
SHS: Secondhand Smoke
THS: Thirdhand Smoke

## 1 Introduction

Tobacco smoke exposure remains a paramount global public health challenge. While the dangers of firsthand and secondhand smoke (SHS) are well‐established, thirdhand smoke (THS) represents a pervasive and emerging environmental toxin (Aurrekoetxea et al. 2013). THS refers to the residual tobacco contamination that adheres to surfaces, dust, and particulate matter long after active smoking has ceased (WHO 2015). This toxic residue, comprising carcinogens like tobacco‐specific nitrosamines (TSNAs), can be re‐emitted into the gas phase, ingested, or absorbed dermally, posing a sustained exposure risk (WHO 2013, Winickoff et al. 2009). Pregnant women and developing fetuses constitute a critically vulnerable population. Exposure to tobacco toxicants is a significant preventable cause of adverse pregnancy outcomes. Globally, it is estimated that over 40% of children are exposed to SHS, contributing to a substantial burden of disease (Jacob et al. 2017). In Iran, despite a low smoking prevalence among women, more than half (56.2%) of pregnant women are exposed to SHS, primarily due to the high prevalence of male smokers in the household (Northrup et al. 2016). THS exposure may pose a particular threat due to its persistent nature and potential for chronic, low‐level exposure in domestic environments where smoking has occurred historically or is permitted occasionally (VW KH‐WR 2019). This is concerning, as studies have linked prenatal exposure to tobacco smoke residues with intrauterine growth restriction, preterm birth, and impaired neurodevelopment (Özpinar et al. 2022, WHO 2011).

Despite this, public awareness and accurate beliefs about THS are markedly low. A significant knowledge gap exists among pregnant women, who often mistakenly believe that ventilating a room or smoking away from others eliminates the risks, leaving them and their unborn children unprotected (WHO 2011, Zhang et al. 2015) gc. The Health Belief Model (HBM) posits that individuals' health‐related actions are influenced by their perceptions of susceptibility, severity, benefits, and barriers, as well as cues to action and self‐efficacy (Matt et al. 2011). Effective health education interventions must therefore not only increase knowledge but also translate it into sustainable protective practices by addressing these underlying beliefs.

While several studies have explored interventions to reduce SHS exposure during pregnancy, research specifically targeting the triad of THS‐related knowledge, beliefs, and practice is strikingly scarce (Sleiman et al. 2010, Matt et al. 2004). Furthermore, the lack of validated, context‐specific tools to measure THS‐related knowledge and practice has hindered robust investigation in this area.

This study was designed to address these critical gaps. We aimed to develop and validate specific measurement tools and then implement a multi‐faceted, theory‐informed educational intervention. The primary objective was to evaluate the efficacy of this intervention in simultaneously improving knowledge, beliefs, and self‐reported protective practices related to THS exposure among pregnant women in Iran.

## 2 Methods

### 2.1 Study Design and Setting

This was a two‐arm, parallel, individually randomized controlled trial. Four urban comprehensive health centers in Isfahan were selected for participant recruitment. Eligible pregnant women were individually randomized to either the intervention or control group using a computer‐generated random number sequence prepared by a statistician not involved in recruitment or intervention delivery. Allocation concealment was maintained using sealed, opaque envelopes.

### 2.2 Participants and Eligibility

Eligible participants were pregnant women in their second or third trimester, aged 18 years or older, literate in Persian, and willing to provide informed consent. Exclusion criteria included having a high‐risk pregnancy condition requiring extensive medical management, plans to relocate before study completion, or concurrent participation in another health intervention study.

### 2.3 Sample Size and Sampling Procedure

The sample size was calculated using G*Power software (version 3.1.9.7). For an independent t‐test, with an alpha of 0.05, power of 80%, and a large effect size (d = 0.8) estimated from a pilot study, a minimum of 42 participants per group was required. Accounting for a potential 15% attrition rate, the final sample size was set at 50 per group (Total N = 100). No design effect or intraclass correlation was applied, as randomization was performed at the individual level.

### 2.4 Data Collection Tools

Data were collected via face‐to‐face interviews by trained research assistants. Due to the nature of the intervention, blinding of outcome assessors was not feasible, which may have introduced social desirability bias. This limitation is addressed in the discussion. Data were collected using four instruments:

1. Demographic and obstetric questionnaire: This researcher‐developed tool collected data on age, education, occupation (of both woman and spouse), economic status (self‐reported as poor, moderate, good, and excellent), housing status, gestational age, parity, history of abortion, and smoking status of the woman and her spouse.
2. Thirdhand smoke knowledge questionnaire (THS‐KQ):  This researcher‐developed instrument consists of 11 items in a True/False/I don't know format, assessing understanding of THS concepts, persistence, exposure pathways, and health effects.

Development and validation: The initial item pool was generated from a literature review. Content validity was assessed quantitatively by a panel of 10 experts using the content validity ratio (CVR) and content validity index (CVI). All items achieved a CVR > 0.62 and a CVI > 0.79. Face validity was established through cognitive interviews with 20 pregnant women.

Construct validity was examined using exploratory factor analysis (EFA) with principal axis factoring and Varimax rotation. The Kaiser–Meyer–Olkin (KMO) measure was 0.82, and Bartlett's test of sphericity was significant (χ^2^ = 345.67, *p* < 0.001), indicating suitability for factor analysis. EFA revealed a one‐factor solution with an eigenvalue of 6.41, explaining 58.3% of the total variance. All items loaded strongly on the single factor (loadings > 0.50).

Reliability was confirmed via test‐retest over a two‐week interval (intraclass correlation coefficient—ICC = 0.86) and good internal consistency (Cronbach's alpha = 0.80). The total score ranges from 0 to 11. The complete item list is provided in Supplementary File 1.

3. Beliefs about thirdhand smoke scale (BATHS): This standardized scale, developed and validated by Haardorfer et al. (2017) and subsequently validated in Turkish and Farsi populations, was used (Yolton et al. 2005, Haardörfer et al. 2017). It consists of 9 items across two subscales: “Persistence of THS” (5 items) and “Harm of THS” (4 items). Responses are recorded on a 5‐point Likert scale from “Strongly Disagree” (1) to “Strongly Agree” (5). The total score ranges from 9 to 45, with higher scores indicating more accurate and stronger beliefs about the risks of THS. In this study, the Farsi version demonstrated excellent reliability (Cronbach's alpha = 0.86).
4. Thirdhand smoke practice questionnaire (THS‐PQ): This researcher‐developed questionnaire contains 9 items with yes/no responses, covering protective behaviors such as avoidance of contaminated spaces, information‐seeking, warning others, and setting household rules.

Development and validation: Similar to the THS‐KQ, content validity was confirmed (CVR > 0.62, CVI > 0.79). Face validity was established through cognitive interviews.

Construct validity was assessed using EFA (principal axis factoring with Varimax rotation). The KMO value was 0.79, and Bartlett's test was significant (χ^2^ = 298.44, *p* < 0.001). EFA yielded a two‐factor solution explaining 61.7% of the total variance:

- Factor 1: avoidance behaviors (items 1, 6, 9)—related to avoiding physical contact with contaminated environments.
- Factor 2: information‐seeking and advocacy (items 2, 3, 4, 5, 7, 8)—related to proactive education and communication.
  All factor loadings exceeded 0.50.

Reliability was supported by high test‐retest correlation (ICC = 0.84) and acceptable internal consistency (Cronbach's alpha = 0.75). The total score ranges from 0 to 9. The full item set is available in Supplementary File 1.

Both researcher‐developed questionnaires were pilot‐tested on a separate sample of 30 pregnant women for clarity, comprehension, and cultural relevance. Cognitive interviews were conducted with 20 pregnant women to ensure item appropriateness and contextual understanding.

Intervention

The intervention was a multi‐component program designed based on the HBM and delivered over several phases, the study detailed protocol has been published previously (Yoshany et al. 2025).

- Phase 1: baseline assessment (pre‐test) (2 weeks): After obtaining written informed consent, all participants in both groups completed the demographic questionnaire, THS‐KQ, BATHS scale, and THS‐PQ via face‐to‐face interviews conducted by trained research assistants.
- Phase 2: intervention implementation (6 weeks): The intervention group received a structured program, while the control group received routine prenatal care (which included general advice on avoiding tobacco smoke but no specific information on THS). The intervention included:
  ○ Four 60 min face‐to‐face group sessions for pregnant women.
  ○ One dedicated 60 min session for spouses.
  ○ Educational pamphlets summarizing key messages.
  ○ Four weeks of reinforcement via daily educational messages and a moderated Q&A group on a popular social media platform (e.g., WhatsApp).
    The content, detailed in Table 1, was designed to address HBM constructs.
- Phase 3: post‐intervention assessment (Post‐test) (2 months after intervention): Two months after the final educational session, all participants in both groups completed the THS‐KQ, BATHS, and THS‐PQ questionnaires again under the same conditions as the baseline.

**TABLE 1:** Structure and content of the educational intervention.

Session	Key content (aligned with HBM constructs)	Methods and materials
1: Understanding THS	‐ Definition and composition of THS vs. SHS. ‐ (Perceived susceptibility): Exposure pathways (inhalation, ingestion and dermal). ‐ (Perceived severity): Persistence on surfaces/dust for months.	‐ Interactive lecture (PPT). ‐ Short animated video. ‐ Q & A.
2: Health impacts	‐ (Perceived severity): Specific risks to fetal development (low birth weight, asthma and preterm birth) and maternal health. ‐ Long‐term consequences for the child.	‐ Case studies/testimonials. ‐ Illustrated pamphlets. ‐ Group discussion on perceived risks.
3: Spousal involvement and communication	‐ (Cues to action, self‐efficacy): Role of partner as a “health ally”. ‐ Strategies to negotiate a completely smoke‐free home/car with family/guests. ‐ Addressing common social barriers.	‐ Role‐playing scenarios. ‐ “Smoke‐Free Home Pledge” cards. ‐ Joint problem‐solving activities for couples.
4: Practical skills and action Plan	‐ (Self‐efficacy): Effective cleaning methods to reduce THS (wet‐wiping and HEPA vacuuming). ‐ Identifying and avoiding exposure hotspots. ‐ (Cues to action): Creating a personal protection plan.	‐ Live demonstration. ‐ Home audit checklist. ‐ Goal‐setting worksheet.
Ongoing reinforcement	‐ (Cues to action): Reinforcing key messages, addressing doubts, and providing motivational support.	‐ Daily educational/text messages for 4 weeks. ‐ Sharing infographics. ‐ Moderated group chat for Q & A.

### 2.5 Data Analysis

Data were analyzed using SPSS Statistics version 23. Descriptive statistics summarized participant characteristics. The Shapiro–Wilk test assessed normality. Baseline comparisons used independent t‑tests and chi‑square tests. Within‑group changes were tested with paired t‑tests (knowledge, beliefs) and the Wilcoxon signed‑rank test (practices). Between‑group outcomes were analyzed using ANCOVA with baseline scores as covariates; assumptions (homogeneity of regression slopes, normality) were verified. For practice scores, which were non‑normal, Quade's nonparametric ANCOVA was applied. Effect sizes (Cohen's d for within‑group, partial eta‑squared for between‑group) are reported. Significance was set at *p* < 0.05.

### 2.6 Ethical Considerations

The study was approved by the Ethics Committee of Shahid Sadoughi University of Medical Sciences, Yazd (Ethics Code: IR.SSU.SPH.REC.1403.032). Written informed consent was obtained from all participants. Confidentiality was assured, and participants were informed of their right to withdraw at any time without any consequence to their medical care.

## 3 Results

### 3.1 Participant Flow and Baseline Characteristics

All 100 recruited participants completed the study (no attrition). Baseline demographic and obstetric characteristics were comparable between the two groups, indicating successful randomization. Independent t‐tests and Chi‐square tests confirmed no statistically significant differences (*p* > 0.05) for all variables, including age, education, economic status, gestational age, and parity (Table 2).

**TABLE 2:** Baseline demographic and obstetric characteristics of participants.

Characteristic	Intervention Group (n = 50)	Control Group (n = 50)	p‐value
Maternal age (years), mean ± SD	32.04 ± 6.08	33.20 ± 5.46	0.31^†^
Gestational age (weeks), mean ± SD	29.85 ± 9.24	28.64 ± 9.18	0.51^†^
Education (woman)	N (%)	N (%)	0.95^‡^
‐ Diploma or less	16 (32%)	17 (34%)	
‐ Bachelor's degree	26 (52%)	26 (52%)	
‐ Master's or higher	8 (16%)	7 (14%)	
Economic status (self‐rated)	N (%)	N (%)	0.57^‡^
‐ Poor/moderate	29 (58%)	33 (66%)	
‐ Good/excellent	21 (42%)	17 (34%)	
Primigravida	17 (34%)	20 (40%)	0.53^‡^
Baseline knowledge score, mean ± SD	13.30 ± 2.68	13.67 ± 3.32	0.52^†^
Baseline beliefs score (BATHS), mean ± SD	25.60 ± 8.12	27.66 ± 8.24	0.21^†^
Baseline practices score, mean ± SD	10.50 ± 1.50	10.88 ± 1.82	0.25^†^

^†^Independent Samples t‐test.

^‡^Chi‐square test.

### 3.2 Primary Outcomes: Knowledge, Beliefs, and Practices

Within‐group analyses using paired t‐tests revealed that the intervention group showed statistically significant improvements in knowledge (t(49) = 9.38, *p* < 0.001) and beliefs (t(49) = 11.34, *p* < 0.001) from baseline to the two‐month follow‐up. The Wilcoxon Signed‐Rank Test showed a significant improvement in self‐reported practices (Z = −8.70, *p* < 0.001). In contrast, the control group showed no significant changes over the same period on any outcome (Knowledge: t(49) = 0.19, *p* = 0.85; Beliefs: t(49) = 1.11, *p* = 0.27; Practices: Z = −1.13, *p* = 0.26) (Table 3).

**TABLE 3:** Within‐group changes in knowledge, beliefs, and practices scores.

Group	Variable	Pre‐test Mean ± SD	Post‐test Mean ± SD	Mean Difference (Post—Pre) [95% CI]	Test Statistic	p‐value	Within‐Group Effect Size (Cohen's d) [95% CI]
Intervention	Knowledge	13.30 ± 2.68	21.52 ± 5.23	8.22 [6.45, 9.99]	t = 9.38	*<0.001	1.32 [0.88, 1.76]
	Beliefs (BATHS)	25.60 ± 8.12	38.46 ± 4.01	12.86 [10.58, 15.14]	t = 11.34	*<0.001	1.60 [1.12, 2.08]
	Practices	10.50 ± 1.50	13.52 ± 2.10	3.02 [2.41, 3.63]	Z = −8.70	*<0.001	1.23 [0.81, 1.65]†
Control	Knowledge	13.67 ± 3.32	13.74 ± 3.60	0.07 [−0.68, 0.82]	t = 0.19	0.85	0.02 [−0.20, 0.24]
	Beliefs (BATHS)	27.66 ± 8.24	28.58 ± 10.55	0.92 [−0.73, 2.57]	t = 1.11	0.27	0.16 [−0.06, 0.38]
	Practices	10.88 ± 1.82	11.10 ± 1.56	0.22 [−0.16, 0.60]	Z = −1.13	0.26	0.12 [−0.08, 0.32]†

Between‐group comparisons using Analysis of Covariance (ANCOVA), controlling for baseline scores, revealed that the intervention had a large and statistically significant effect on post‐intervention knowledge (F(1, 97) = 179.61, *p* < 0.001), beliefs (F(1, 97) = 77.52, *p* < 0.001), and practices (F(1, 97) = 54.14, *p* < 0.001). The intervention group had significantly higher adjusted mean scores on all outcomes compared to the control group.

## 4 Discussion

This randomized controlled trial provides robust evidence that a structured, multi‐component educational intervention, grounded in the Health Belief Model, can effectively and simultaneously improve knowledge, beliefs, and self‐reported protective practices regarding thirdhand smoke among pregnant women. The significant and substantial improvements observed in the intervention group across all three domains, contrasted with the stability of scores in the control group, underscore the efficacy of the intervention.

The dramatic increase in knowledge scores highlights the success of the intervention in addressing a critical information deficit about THS. Our findings align with studies that have used educational interventions for SHS, such as Inaoka et al. (2023) who used leaflets, and Abu‐bakar et al. (2022) who utilized mobile health platforms (Öberg et al. 2011, Alghamdi et al. 2016). However, our study extends this evidence by demonstrating that a comprehensive, THS‐specific curriculum, delivered through a blended approach of interactive group sessions, visual aids, and sustained virtual reinforcement, is exceptionally effective in conveying the complex and often intangible nature of THS. This multi‐modal approach likely catered to diverse learning styles and reinforced key messages more effectively than a single‐mode delivery.

The profound positive shift in beliefs, as measured by the validated BATHS scale, is a cornerstone finding of this study. Beliefs are cognitive constructs that are often resistant to change. The success of our intervention can be directly attributed to its firm theoretical foundation in the HBM. By meticulously designing content to enhance perceived susceptibility (e.g., clarifying all exposure pathways) and perceived severity (e.g., detailing specific risks to fetal neurodevelopment), the intervention facilitated a fundamental cognitive restructuring regarding the threat of THS. This result resonates with the work of Cardirci et al. (2021) and Özpinar et al. (2022), who established that THS beliefs are modifiable and are influenced by education and exposure to information (Haardörfer et al. 2017, Wahabi et al. 2013). Our intervention demonstrates that targeted education can proactively cultivate accurate and protective beliefs, potentially mitigating the influence of lower baseline education levels or pre‐existing misconceptions.

The significant improvement in self‐reported practices indicates that the intervention successfully bridged the gap between increased knowledge/modified beliefs and tangible action—a transition often regarded as the most significant challenge in health promotion. This translation was likely driven by the intervention's strong emphasis on building self‐efficacy through hands‐on, practical skill‐building sessions (e.g., cleaning demonstrations and creating personalized action plans) and providing potent cues to action (e.g., daily reminder messages, a physical “Smoke‐Free Home Pledge”). Our results corroborate findings from Moradi et al. (2019), who used family counseling based on the BASNEF modelshowing that reinforcing practical strategies is key to behavior change (Aurrekoetxea et al. 2014, Baheiraei et al. 2012). A pivotal strength of our intervention was the deliberate involvement of spouses, which aimed to create a supportive home environment conducive to enacting and maintaining new behaviors, a factor critically identified by Malek et al. (2010) (Malek et al. 2010).

The effectiveness of our intervention is consistent with previous research focusing on spousal education. Studies by Karimiankakolaki and colleagues demonstrated that educational programs for male smokers could significantly improve their knowledge, attitude, and self‐efficacy regarding secondhand smoke exposure during their wives' pregnancies (Karimiankakolaki and Mazloomy Mahmoodabad 2023, Karimiankakolaki et al. 2023). Our study extends these findings by shifting the focus from secondhand to thirdhand smoke and by directly empowering pregnant women themselves, rather than their smoking spouses. Furthermore, the rigorous development process of our intervention aligns with the protocol recommended by Karimiankakolaki et al. (2019), emphasizing the importance of theory‐based, needs‐assessed educational design. However, while their interventions successfully improved male smokers' self‐efficacy and behavior, they noted that changes in perceived susceptibility and severity were less significant. In contrast, our women‐centric approach demonstrated substantial improvements in these critical belief domains regarding THS, suggesting that direct education of the at‐risk individual may be particularly effective for enhancing risk perception of this less visible environmental threat.

When viewed holistically, the synergistic improvement across all three domains underscores the power of a comprehensive, theory‐driven approach. The intervention did not merely act as an information delivery system; it created an experiential learning journey that fostered belief modification and equipped participants with the practical tools and confidence (self‐efficacy) to implement change. This integrated approach addresses a common limitation noted in studies like that of Al‐Mohamed et al. (2021) in Saudi Arabia, where knowledge improved without a corresponding shift in behavior, possibly due to an insufficient focus on self‐efficacy and environmental support structures (MazloomyMahmoodabad et al. 2019).

### 4.1 Strengths

Key strengths of this study include its rigorous randomized controlled trial design, successful randomization ensuring baseline comparability, perfect retention (100%), the development and validation of context‐specific measures for knowledge and practice, and the delivery of a comprehensive, theory‐based, multi‐component intervention. The active involvement of spouses further enhanced the intervention's ecological relevance and potential for sustainable impact.

### 4.2 Limitations

Several limitations should be acknowledged. First, the two‐month follow‐up period, while sufficient for assessing initial impact, is too short to evaluate the long‐term sustainability of behavioral changes. Second, reliance on self‐reported practices remains susceptible to social desirability bias, despite the use of validated instruments. Third, the study was conducted in a single city in Iran with participants recruited from selected urban health centers, which may limit the generalizability of findings to other cultural, socioeconomic, or healthcare settings. Fourth, although spousal involvement was incorporated and attendance was high, its specific quantitative contribution to outcomes was not examined through formal mediation analysis.

### 4.3 Future Directions

Future research should incorporate extended follow‐up periods (e.g., 6–12 months) to evaluate the durability of intervention effects. The inclusion of objective biochemical measures, such as dust nicotine or surface wipe analyses, is recommended to complement self‐reported data and reduce social desirability bias. Multicenter trials across diverse geographic and socioeconomic settings would enhance external validity. Additionally, studies with larger samples should conduct formal mediation analyses to quantify the role of spouse engagement and other potential mechanisms of change. Finally, cost‐effectiveness analyses would be valuable to inform the scalability and integration of such interventions into routine prenatal care.

## 5 Conclusion

In conclusion, this study provides compelling and holistic evidence that a structured, multi‐component educational intervention can significantly enhance pregnant women's understanding, beliefs, and self‐reported protective behaviors regarding thirdhand smoke. By successfully addressing the complete pathway from knowledge acquisition and belief modification to the enactment of protective practices, such programs offer a powerful and essential strategy to safeguard maternal and fetal health from this persistent and underestimated environmental hazard. The findings strongly advocate for the systematic integration of these evidence‐based educational modules into standard prenatal care protocols. This represents a feasible, cost‐effective, and highly impactful public health initiative with the potential to reduce the burden of disease associated with tobacco smoke exposure in a vulnerable population, contributing to the broader goals of maternal and child health promotion.

## Author Contributions

All authors were involved in study conception, design, drafting of the manuscript, N.Y, Z.A, M.K, S.J, A.G, and Z.K were involved in write and revise the manuscript. All authors have read and approved the final version of the manuscript.

## Funding

This research protocol was supported by the Shahid Sadoughi University of Medical Sciences, Yazd, Iran.

## Ethics Statement

Ethical approval for this study has been obtained by the ethics committee affiliated with Shahid Sadoughi University of Medical Sciences, Yazd, Iran (reference number IR.SSU.SPH.REC.1403.032) and Informed consent was obtained from all study participants. This study adhered to the Declaration of Helsinki in this regard.

## Supporting information




**Supplementary File 1**. Item lists of the Thirdhand Smoke Knowledge Questionnaire (THS‐KQ) and Thirdhand Smoke Practice Questionnaire (THS‐PQ).

## Data Availability

The datasets generated and/or analyzed during the current study are not publicly available due [due to privacy and confidentiality agreements with the participants] but are available from the corresponding author on reasonable request.

## References

[brb371330-bib-0028] Abu‐Baker, N. N. , A.l‐J. IA , N. M. Al‐Ali , and M. A Al‐Khalaileh . 2022. “The Effect of Health Education on Second‐hand Smoke Knowledge and Exposure Among Pregnant Women in Jordan.” International Journal of Environmental Research and Public Health 19, no. 18: 11668.36141945

[brb371330-bib-0029] AL‐Mohammad, F. , and D Youssef Wazqar . 2021. “Factors Associated With Self‐management Practices and Self‐efficacy Among Adults With Cancer Under Treatment in Saudi Arabia.” Journal of Clinical Nursing 30, no. 21‐22: 3301–3331. 33963631 10.1111/jocn.15843

[brb371330-bib-0001] Alghamdi, A. , H. Jokhadar , I. Alghamdi , S. Abdullah , O. Alsohibani , and H. Wahabi . 2016. “Socioeconomic Determinants of Exposure to Secondhand Smoke Among Pregnant Women.” International Journal of Womens Health and Reproduction Sciences 4, no. 2: 59–63. 10.15296/ijwhr.2016.14.

[brb371330-bib-0003] Aurrekoetxea, J. J. , M. Murcia , M. Rebagliato , et al. 2014. “Factors Associated With Second‐Hand Smoke Exposure in Non‐Smoking Pregnant Women in Spain: Self‐Reported Exposure and Urinary Cotinine Levels.” Science of the Total Environment 470–471: 1189–1196. 10.1016/j.scitotenv.2013.10.110. 24246942

[brb371330-bib-0002] Aurrekoetxea, J. J. , M. Murcia , M. Rebagliato , et al. 2013. “Determinants of Self‐Reported Smoking and Misclassification During Pregnancy, and Analysis of Optimal Cut‐Off Points for Urinary Cotinine: A Cross‐Sectional Study.” BMJ Open 3: e002034. 10.1136/bmjopen-2012-002034. PMC3563144 23355667

[brb371330-bib-0004] Baheiraei, A. , R. Faghihi , A. Mirmohammad , and N. Kazem . 2012. “Predictors of Home Smoking Ban in Households in Pregnant Women.” Payesh 11, no. 4: 511–517. http://payeshjournal.ir/article‐1‐447‐en.html.

[brb371330-bib-0030] Çadirci, D. , S. Özpinar , Ü. Özbey , and Ö. S Gümüş . 2021. “Validity and Reliability of Turkish Version of Beliefs about Third‐Hand Smoke Scale.” Central European Journal of Public Health 29, no. 1: 56–56. 33831287 10.21101/cejph.a6578

[brb371330-bib-0005] Haardörfer, R. , C. Berg , C. Escoffery , Ł. Bundy , M. Hovell , and M. Kegler . 2017. “Development of a Scale Assessing Beliefs about ThirdHand Smoke (BATHS).” Tobacco Induced Diseases 15: 4. 10.1186/s12971-017-0112-4. 28104999 PMC5240270

[brb371330-bib-0031] Inaoka, K. , T. Kawai , D. B. Trisnaningtyas , et al. 2023. “Effects of a Comic Booklet Intervention on Intention and Behavior for Reducing Secondhand Smoke Exposure in Pregnant Women.” Healthcare 11, no. 23: 3061. 38063629 10.3390/healthcare11233061 PMC10706841

[brb371330-bib-0006] Jacob, P. , N. L. Benowitz , H. Destaillats , et al. 2017. “Thirdhand Smoke: New Evidence, Challenges, and Future Directions.” Chemical Research in Toxicology 30, no. 1: 270–294. 10.1021/acs.chemrestox.6b00343. 28001376 PMC5501723

[brb371330-bib-0007] Karimiankakolaki, Z. , S. Gerayllo , and S. Mazloomy Mahmoodabad . 2023. “The Effect of Training Male Smokers About the Effects of Secondhand Smoke in Pregnant Wives on Their Self‐Efficacy.” Journal of Family and Reproductive Health 17, no. 4: 250. 10.18502/jfrh.v17i4.14597. 38807626 PMC11128728

[brb371330-bib-0008] Karimiankakolaki, Z. , S. S. Mazloomy Mahmoodabad , and A. Kazemi . 2023. “Designing, Implementing and Evaluating an Educational Program Regarding the Effects of Second‐Hand Smoke in Pregnancy on the Knowledge, Attitude and Performance of Male Smokers.” Reproductive Health 20, no. 1: 82. 10.1186/s12978-023-01630-y. 37277775 PMC10240778

[brb371330-bib-0009] Karimiankakolaki, Z. , S. S. Mazloomy Mahmoodabad , A. Kazemi , and H. Fallahzadeh . 2019. “Designing an Educational Intervention on Second‐Hand Smoke in Smoker Men on the Exposure of Pregnant Wives: A Protocol for a Randomized Controlled Tria.” Reproductive Health 16, no. 1: 11. 10.1186/s12978-019-0673-1. 30709402 PMC6359865

[brb371330-bib-0010] Malek, M. , R. Eskandarian , and R. Ghorbani . 2010. “Epidemiology of Smoking Among Adult Women Population of Semnan Province.” Koomesh: Journal of Semnan University of Medical Sciences 11, no. 2: 75–82. https://www.sid.ir/paper/37195/fa.

[brb371330-bib-0011] Matt, G. , P. Quintana , M. Hovell , et al. 2004. “Households Contaminated by Environmental Tobacco Smoke: Sources of Infant Exposures.” Tobacco Control 13, no. 11: 29–37. 10.1136/tc.2003.003889. 14985592 PMC1747815

[brb371330-bib-0012] Matt, G. E. , P. J. E. Quintana , H. Destaillats , et al. 2011. “Thirdhand Tobacco Smoke: Emerging Evidence and Arguments for a Multidisciplinary Research Agenda.” Environmental Health Perspectives 119, no. 9: 1218–1226. 10.1289/ehp.1103500. 21628107 PMC3230406

[brb371330-bib-0013] MazloomyMahmoodabad, S. , Z. Karimiankakolaki , A. Kazemi , N. Mohammadi , and H. Fallahzadeh . 2019. “Exposure to Secondhand Smoke in Iranian Pregnant Women at Home and the Related Factors.” Tobacco Prevention and Cessation 5, no. 7: 1–9. 10.18332/tpc/104435. 32411872 PMC7205101

[brb371330-bib-0032] Moradi, F. , A. Khosravi , and S Hosseinzadeh . 2019. “The Effectiveness of family Counselling on Reducing Exposure to Secondhand Smoke Among Pregnant Women in Iran.” Tob Prev Cessat 5: 37. 32411903 10.18332/tpc/113105 PMC7205145

[brb371330-bib-0014] Northrup, T. F. , P. Jacob , N. L. Benowitz , et al. 2016. “Thirdhand Smoke: State of the Science and a Call for Policy Expansion.” Public Health Reports 131, no. 2: 233–238. 10.1177/003335491613100206. 26957657 PMC4765971

[brb371330-bib-0016] Özpinar, S. , Y. Demir , B. Yazicioğlu , S. Bayçelebi , and B. Yazicioğlu . 2022. “Pregnant Women's Beliefs About Third‐Hand Smoke and Exposure to Tobacco Smoke.” Central European Journal of Public Health 30, no. 3: 186–191. 10.21101/cejph.a7063. 36239362

[brb371330-bib-0015] Öberg, M. , M. Jaakkola , A. Woodward , A. Peruga , and A. Prüss‐Ustün . 2011. “Worldwide Burden of Disease From Exposure to Second‐Hand Smoke: A Retrospective Analysis of Data From 192 Countries.” The Lancet 377, no. 9760: 139–146. 10.1016/S0140-6736(10)61388-8. 21112082

[brb371330-bib-0017] Sleiman, M. , L. Gundel , J. Pankow , I. P. Jacob , B. Singer , and H. Destaillats . 2010. “Formation of Carcinogens Indoors by Surface‐Mediated Reactions of Nicotine With Nitrous Acid, Leading to Potential Thirdhand Smoke Hazards.” Proceedings of the National Academy of Sciences 107, no. 15: 6576–6581. 10.1073/pnas.0912820107. PMC2872399 20142504

[brb371330-bib-0018] VW KH‐WR . 2019. “Third‐Hand Smoke (THS): What Is It and What Should We Do About It.” Journal of the Formosan Medical Association 118, no. 11: 1478–1479. 10.1016/j.jfma.2019.08.025. 31500939

[brb371330-bib-0021] *WHO* . 2011. Gender, Health, Tobacco and Equity. http://www.who.int/tobacco/publications/gender/gender_tobacco_2010.pdf.

[brb371330-bib-0022] *WHO* . 2010. Worldwide Burden of Disease From Exposure to Second‐Hand Smoke. http://www.who.int/quantifying_ehimpacts/publications/shsarticle2010/en/. 10.1016/S0140-6736(10)61388-8 21112082

[brb371330-bib-0023] *WHO* . 2013. Secondhand Smoke (SHS) Facts. https://www.who.int/tobacco/publications/second_hand/factsheet/en/.

[brb371330-bib-0024] *WHO* . 2015. Tobacco Free Initiative (TFI) Second‐Hand Tobacco Smoke. https://www.who.int/tobacco/publications/second_hand/en/.

[brb371330-bib-0019] Wahabi, H. A. , R. A. Alzeidan , A. A. Fayed , A. Mandil , G. Al‐Shaikh , and S. A. Esmaeil . 2013. “Effects of Secondhand Smoke on the Birth Weight of Term Infants and the Demographic Profile of Saudi Exposed Women.” BMC Public Health [Electronic Resource] 13, no. 1: 341. 10.1186/1471-2458-13-341. 23587116 PMC3641009

[brb371330-bib-0020] Winickoff, J. P. , J. Friebely , S. E. Tanski , et al. 2009. “Beliefs About the Health Effects of “Thirdhand” Smoke and Home Smoking Bans.” Pediatrics 123, no. 1: e74–e79. 10.1542/peds.2008-2184. 19117850 PMC3784302

[brb371330-bib-0025] Yolton, K. , K. Dietrich , P. Auinger , B. P. Lanphear , and R. Hornung . 2005. “Exposure to Environmental Tobacco Smoke and Cognitive Abilities Among US Children and Adolescents.” Environmental Health Perspectives 113, no. 1: 98–103. 10.1289/ehp.7210. 15626655 PMC1253717

[brb371330-bib-0026] Yoshany, N. , Z. Karimiankakolaki , M. Khodayarian , S. Jambarsang , A. Ghaderi , and Z. Afkhami . 2025. “Invisible Smoke, Real Threat: Can Education Protect Pregnant Women From Thirdhand Smoke Exposure?(A Protocol Study).” BMC Pregnancy and Childbirth 25, no. 1: 1117. 10.1186/s12884-025-08215-6. 41120919 PMC12539110

[brb371330-bib-0027] Zhang, L. , J. Hsia , X. Tu , et al. 2015. “Exposure to Secondhand Tobacco Smoke and Interventions Among Pregnant Women in China: A Systematic Review.” Preventing Chronic Disease 12, no. 35: 11–11. 10.5888/pcd12.140377. PMC4372160 25789496

