# Supplementary material for: The Effectiveness of a Structured Educational Intervention on Knowledge, Beliefs, and Self‐Reported Practices of Pregnant Women Exposed to Thirdhand Smoke: A Randomized Controlled Trial
Source: Brain Behav. 2026 Jun 8;16(6):e71330. doi: 10.1002/brb3.71330 (PMC13244072; doi:10.1002/brb3.71330)
Supplement: Supplementary file 1 — Item lists of the Thirdhand Smoke Knowledge Questionnaire (THS‐KQ) and Thirdhand Smoke Practice Questionnaire (THS‐PQ). [file BRB3-16-e71330-s001.docx]

**Supplementary File 1: Item Lists of the THS Knowledge and Practice Questionnaires**

#### ****A. Thirdhand Smoke Knowledge Questionnaire (THS-KQ)****

- **Number of items:** 11
- **Response format:** True / False / I don’t know
- **Scoring:** Correct = 1, Incorrect/Don’t know = 0 | Total score range: 0–11
- **Items (English translation):**

1. Deposition of smoke particles and toxins on surfaces, especially in enclosed spaces, is harmful to everyone.
2. A room where smoking occurred but no smoke odor remains is still a threat to you.
3. A room where smoking occurred previously but windows/doors are open still poses a contamination risk.
4. If your spouse smokes outside, their clothing poses a risk when they enter the home.
5. THS in a room can cause low birth weight in newborns if a pregnant woman is exposed.
6. If someone smoked in a room, you can be exposed to harmful effects simply by breathing there.
7. If someone smoked in a room, you can be exposed to harmful effects by touching surfaces.
8. A child crawling on a carpet in a room where smoking occurred can suffer health effects.
9. A child touching surfaces and hand-to-mouth contact in a room where smoking occurred can cause health effects.
10. Pregnant women’s exposure to THS poses pregnancy health risks.
11. Pregnant women’s exposure to THS increases the risk of preterm birth.

#### ****B. Thirdhand Smoke Practice Questionnaire (THS-PQ)****

- **Number of items:** 9
- **Response format:** Yes / No
- **Scoring:** Yes = 1, No = 0 | Total score range: 0–9
- **Items (English translation):**

1. I avoid riding in a smoker’s car whenever possible.
2. I seek and apply scientific information about the harms of THS.
3. I warn other pregnant women about the risks of THS.
4. I read scientific brochures about THS effects.
5. I recommend scientific brochures about THS to others.
6. I avoid touching surfaces in public places or homes of smokers.
7. I remind household members and guests not to smoke in my personal car.
8. I remind household members and guests not to smoke in my personal home.
9. I prevent my child from contacting surfaces in public places or spaces where smoking has occurred.
